# Toward Respiratory Assessment Using Depth Measurements from a Time-of-Flight Sensor

**DOI:** 10.3389/fphys.2017.00065

**Published:** 2017-02-07

**Authors:** Charles Sharp, Vahid Soleimani, Sion Hannuna, Massimo Camplani, Dima Damen, Jason Viner, Majid Mirmehdi, James W. Dodd

**Affiliations:** ^1^Academic Respiratory Unit, University of BristolBristol, UK; ^2^Faculty of Engineering, University of BristolBristol, UK; ^3^North Bristol NHS Trust, North Bristol Lung CentreBristol, UK

**Keywords:** respiratory function tests, spirometry, home monitoring

## Abstract

**Introduction:** There is increasing interest in technologies that may enable remote monitoring of respiratory disease. Traditional methods for assessing respiratory function such as spirometry can be expensive and require specialist training to perform and interpret. Remote, non-contact tracking of chest wall movement has been explored in the past using structured light, accelerometers and impedance pneumography, but these have often been costly and clinical utility remains to be defined. We present data from a 3-Dimensional time-of-flight camera (found in gaming consoles) used to estimate chest volume during routine spirometry maneuvres.

**Methods:** Patients were recruited from a general respiratory physiology laboratory. Spirometry was performed according to international standards using an unmodified spirometer. A Microsoft Kinect V2 time-of-flight depth sensor was used to reconstruct 3-dimensional models of the subject's thorax to estimate volume-time and flow-time curves following the introduction of a scaling factor to transform measurements to volume estimates. The Bland-Altman method was used to assess agreement of model estimation with simultaneous recordings from the spirometer. Patient characteristics were used to assess predictors of error using regression analysis and to further explore the scaling factors.

**Results:** The chest volume change estimated by the Kinect camera during spirometry tracked respiratory rate accurately and estimated forced vital capacity (FVC) and vital capacity to within ± <1%. Forced expiratory volume estimation did not demonstrate acceptable limits of agreement, with 61.9% of readings showing >150 ml difference. Linear regression including age, gender, height, weight, and pack years of smoking explained 37.0% of the variance in the scaling factor for volume estimation. This technique had a positive predictive value of 0.833 to detect obstructive spirometry.

**Conclusion:** These data illustrate the potential of 3D time-of-flight cameras to remotely monitor respiratory rate. This is not a replacement for conventional spirometry and needs further refinement. Further algorithms are being developed to allow its independence from spirometry. Benefits include simplicity of set-up, no specialist training, and cost. This technique warrants further refinement and validation in larger cohorts.

## Introduction

Rising living standards and advances in healthcare mean that people are living longer, often with more than one long term medical condition. The resulting rise in demand on healthcare systems has led to an increasing interest in technologies that may enable remote monitoring of long term conditions in the home environment (De San Miguel et al., 2013; Chatwin et al., 2016). Respiratory diseases such as chronic obstructive pulmonary disease are one of the most common long term conditions. It is thought to effect around 3 million people in the UK alone and accounts for over 1 billion pounds a year in healthcare expenditures and is estimated to become the third leading cause of death. The current gold standard physiological measure of lung function is spirometry; this requires the subject to co-ordinate a forced exhalation into a pneumotachygraph which produces a flow volume loop. Spirometry is expensive, requires specialist equipment and training to perform and interpret and not everyone is able to perform the procedure (very young, frail, or cognitively impaired). A novel approach to respiratory function assessment which has potential to improve the quality of disease monitoring is non-contact measurements of respiratory motion.

Non-contact lung volume measurement has been explored previously including structured light (Morgan et al., 1984; Lau et al., 2009; Aoki et al., 2012), accelerometers (Reich and McHenry, 1990), optoelectric plethysmography (Cala et al., 1996; Aliverti et al., 2001), and impedance pneumography (Sackner et al., 1980). These novel technologies could also be used for real-time home monitoring of long term conditions, supporting self-management and preventing adverse health events such as hospitalizations.

Other possible applications include, population screening for lung disease, gating controls for radiological imaging techniques (such as magnetic resonance imaging, MRI) and patient-ventilator synchronization for non-invasive or invasive mechanical ventilation. The techniques assessed to date have been costly and their role in day to day practice remains to be defined.

Recent technological advances have reduced the cost of time-of-flight cameras, which measure 3D (3-dimensional) movement in real-time, from which motion can be detected to within 0.1 mm (Penne et al., 2008). This is achieved through calculation of multiple distances between the camera, the object of interest and a virtual plane a specified distance from the camera using Light Imaging, Detection, And Ranging (LIDAR) technology, a detection system which works on the principle of radar, but uses light from a laser.

We present data from a developmental study designed to understand technology used in gaming consoles as a means of non-contact monitoring of breathing in a large real world clinical population. This study objective was to test the hypothesis that respiratory monitoring using time-of-flight technology could give accurate estimates of lung volumes with both forced and relaxed spirometric maneuvres. This technique does not aim to replace spirometry for clinical measurements, but may be able to take a complementary role in respiratory monitoring, in addition to the potential applications suggested above.

## Methods

### Study design and subjects

In this prospective, observational study, adult patients referred for spirometry for clinical indications were recruited from North Bristol respiratory physiology laboratory, U.K. Inclusion criteria were referral for spirometry and age over 18 years. Patients unable to provide informed consent or perform spirometry were excluded. Ethical approval for the study was granted (North West England Research Ethics Committee reference–15/NW/0040). Full informed consent; participants signed consent forms after review of patient information documents.

### Equipment set-up

The Kinect v2 (Microsoft, Redmond, WA, USA) is the state-of-the-art consumer available depth sensor originally produced to be used with the Microsoft Xbox One gaming console. However, since it is an inexpensive and publicly available depth sensor, it has been exploited in numerous applications and its performance and accuracy has been assessed in several statistical analysis and studies (Lachat et al., 2015; Pagliari and Pinto, 2015).

The Kinect v2 is able to measure the distance between the sensor and surface of objects in the sensor's field of view, using Time-of-Flight technology. 3D model of the objects of the scene can be generated by using the Kinect camera parameters, i.e., focal length and principal point. The subject's respiratory function can be estimated by volume-time curves created by the Kinect depth measurements. These curves are created by (a) reconstructing 3D model of the subject's chest, and (b) estimating its volume as a function of time for the whole sequence.

**Figure 1:**
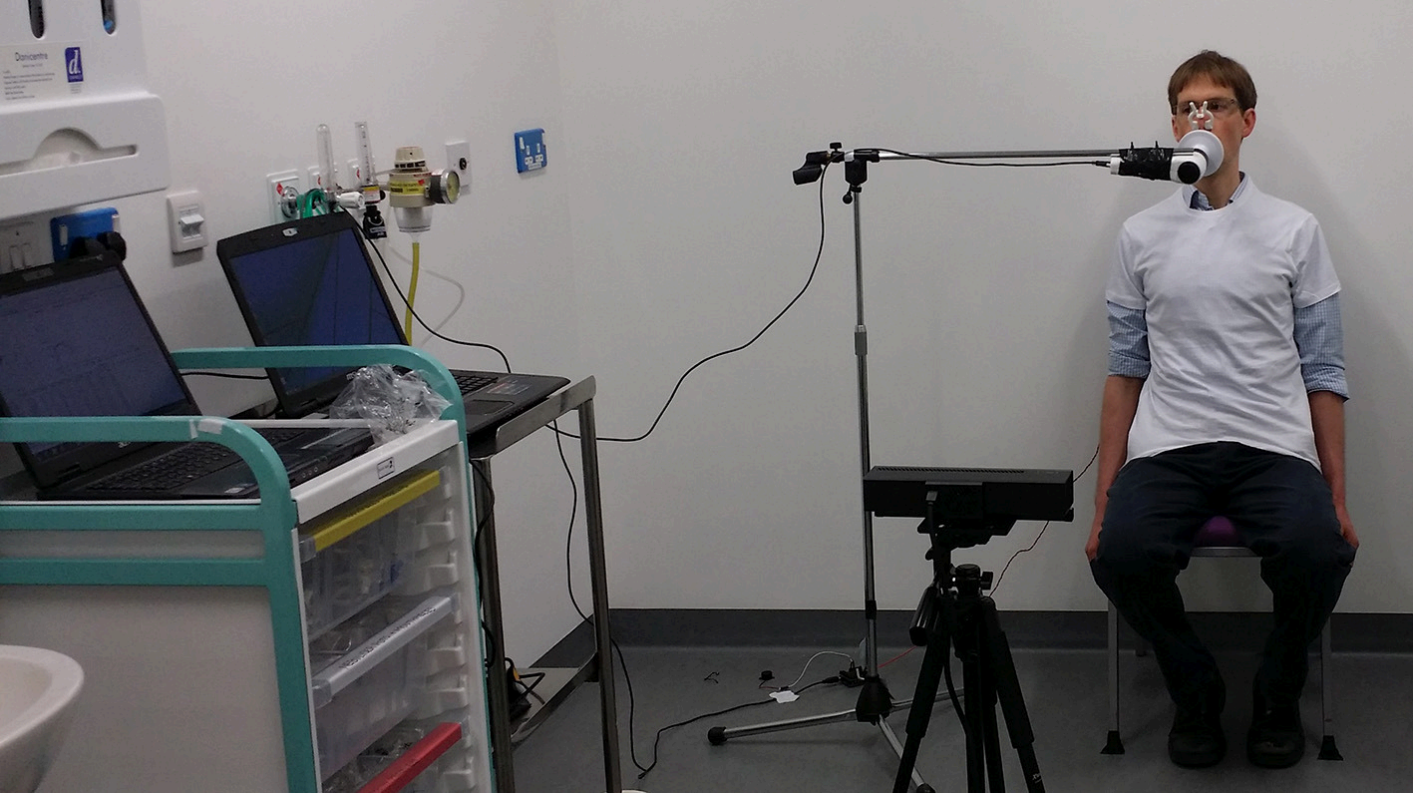
**Demonstration of the equipment configuration for the study, including spirometer, Kinect. and the subject**.

Subjects were seated upright with their arms by their sides on a normal chair with no armrests, facing a Kinect v2 positioned 1.5 m from the subject, at a height of 0.6 m. Patients were provided with figure-hugging t-shirts to improve accuracy of chest motion tracking (Figure 1). Spirometry maneuvres were performed according to American Thoracic Society/European Respiratory Society (ATS/ERS) standards (Miller et al., 2005) while simultaneously recording with the camera and an unmodified spirometer (nSpire Health, Longmont, CO, USA), on a mounting to keep tubing away from the chest wall. Spirometric measures recorded were forced vital capacity (FVC), forced expiratory volume in 1 s (FEV1), relaxed vital capacity (VC) and inspiratory capacity (IC). The respiratory rate was calculated from the resting respiratory cycle, defined as the number of cycles occurring in 1 min.

Following a period of normal breathing to the resting respiratory cycle, relaxed, and FVC maneuvres were performed. At least three efforts were made for each of the relaxed and forced capacity maneuvres (see online video, https://youtu.be/AX4BvyoKYYQ). The total time for the procedure was no longer than that required for normal spirometry, after the subjects had put on the t-shirt.

### Model construction and volume estimation

**Figure 2:**
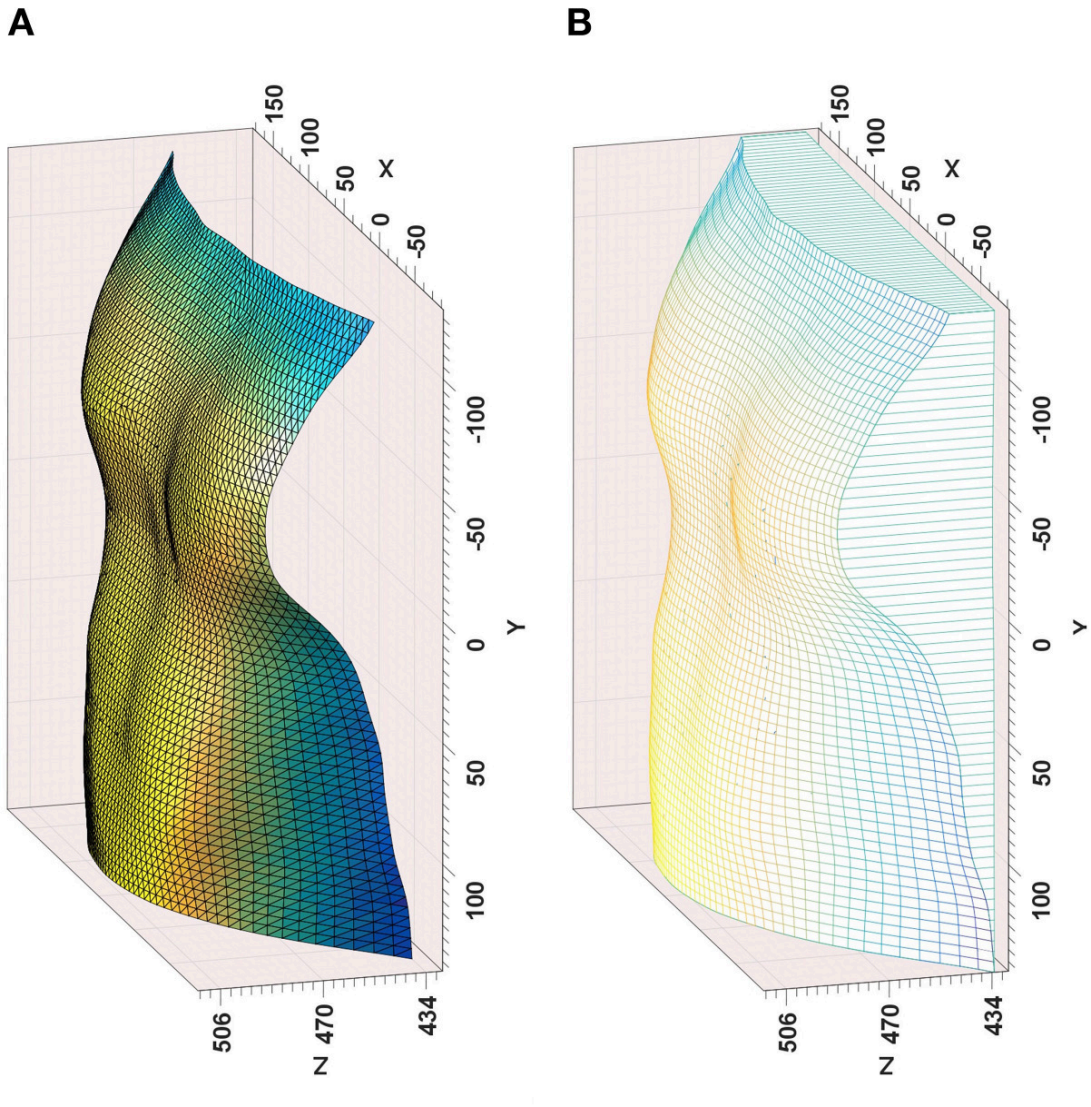
**Outputs from the time-of-flight camera modeling—(A)** Reconstructed chest surface, **(B)** Volume estimation from reference plane, including lateral chest wall.

After segmenting subject's chest using the skeleton joint data provided by the Kinect, a point cloud of the thoracic wall was then generated and triangulated (de Berg et al., 2008). The subject's posterior chest wall cannot be used to estimate the thoracic wall volume using Divergence Theorem, therefore this volume is estimated by considering a reference plane at a predefined distance from the subject's back and also the lateral chest wall as shown in Figure 2. The change in volume of the chest is key to estimating the volume-time curve, therefore the position of the reference plane is not important. The volume of the thoracic wall (*V*_*t*_) is estimated by integrating the volume between each partial triangle and the reference plane:

(1) $$\begin{array}{r} V_{t}=\overset{N_{t}}{\underset{i=1}{\sum }}\left(\frac{1}{6}\overset{3}{\underset{j=1}{\sum }}z_{ij}\times |\begin{array}{ccc} x_{i1} & y_{i1} & 1\\ x_{i2} & y_{i2} & 1\\ x_{i3} & y_{i3} & 1 \end{array}|\right), \end{array}$$

where *(x*_*ij*_*, y*_*ij*_*, z*_*ij*_*)* denotes vertex *j* of triangle *i* and *N*_*t*_ is the total number of triangles. Technical details for the model construction have been reported elsewhere (Soleimani et al., 2015, 2016).

**Figure 3:**
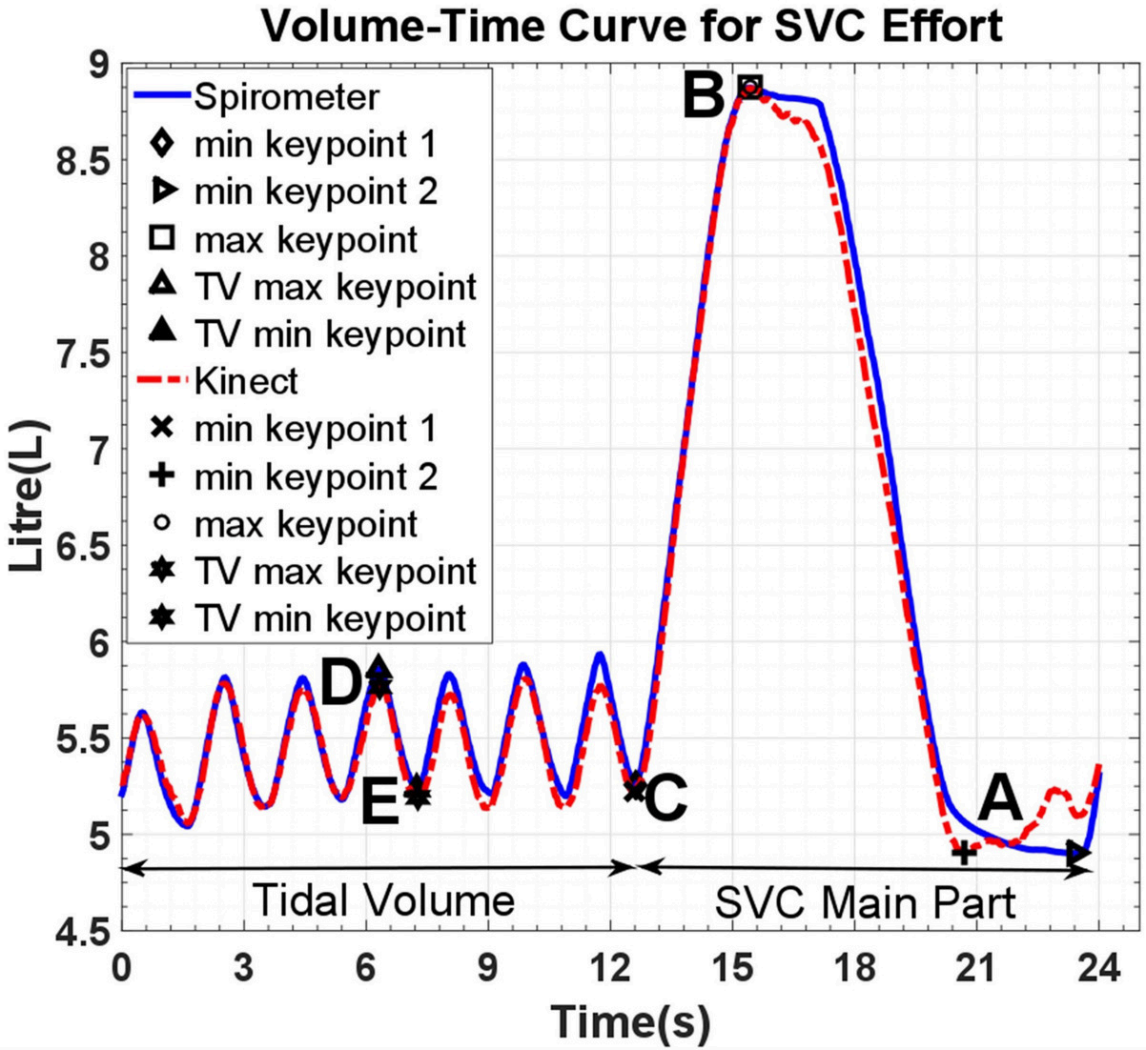
**Volume-Time curve of spirometer and model estimates; reference points are for tidal volume (D–E), inspiratory capacity (C–B), and vital capacity (B–A)**. SVC, Slow Vital Capacity; TV, Tidal volume.

The beginning and end of respiratory maneuvres were estimated using a two stage procedure. A button is pressed at the beginning and end of maneuvre, providing an estimation of the beginning and end of the respiratory maneuvre. To be able to evaluate the method results, spirometer and Kinect volume-time curves must be temporally aligned precisely. The similarity between the spirometer and Kinect volume-time curves (especially in the first normal breathing cycles) enables us to apply a normalized windowed cross correlation to find the delay between two curves and align them, thereby detecting the beginning and end of the maneuvre (Figure 3). Following this procedure to produce volume-time curves, volumes for spirometry maneuvres could be estimated and respiratory rate assessed from the tracing of the respiratory cycle.

Although the Kinect volume-time curve is computed by estimating the volume of the 3D reconstructed thoracic wall as a function of time, it is originally based on the changes in distance of subject's chest surface from the sensor and therefore does not measure the chest volume in liters. Thus, it must be calibrated to reflect the chest volume change. This calibration is performed by computing scaling factors through a linear regression analysis of the Kinect volume-time curve and its spirometer counterpart using specific key points which are automatically extracted from the curves (Figure 3).

### Statistical analysis

Statistical analysis was performed using Stata v14.1 statistical software (StataCorp LP, Texas, USA). Descriptive statistics were used to summarize patient characteristics and spirometry data. Correlation coefficients were used to demonstrate the relationship between model chest volume estimation and spirometric measures.

The Bland-Altman method was used to compare values estimated for measures of interest with those generated by concurrent spirometry. As per international guidance, a difference of 150 ml was defined as the acceptable limit, allowing for use of the Bland Altman method with non-parametric data (Bland and Altman, 1999; Miller et al., 2005). The absolute mean difference (bias) between techniques is shown with standard deviation (*SD*) and the percentage of values outside the 150 ml limits of agreement. Values were not log transformed and relative percentages were not used (Bland and Altman, 1999; Dewitte et al., 2002). Within patient variability was calculated by the coefficient of variance (CV).

A multivariable linear regression model was used to identify variables predictive of error between spirometric and depth-sensor estimated measurements. Variables were included where they are known to influence spirometry values.

To further investigate the relationships between the model estimates and spirometry measures, the scaling factors were analyzed using a multivariable regression model including variables known to influence spirometry values.

### Study funding

This work was part of an interdisciplinary collaboration between clinicians and engineers from the ≤ 15 million Engineering and Physical Sciences Research Council (EPSRC) funded SPHERE project (Sensor Platform for Healthcare in Residential Environment, http://www.irc-sphere.ac.uk/). SPHERE is developing a number of different sensors that will combine to build a picture of how we live in our homes and to diagnose and manage health conditions (Zhu et al., 2015).

## Results

### Study subjects

**Figure 4:**
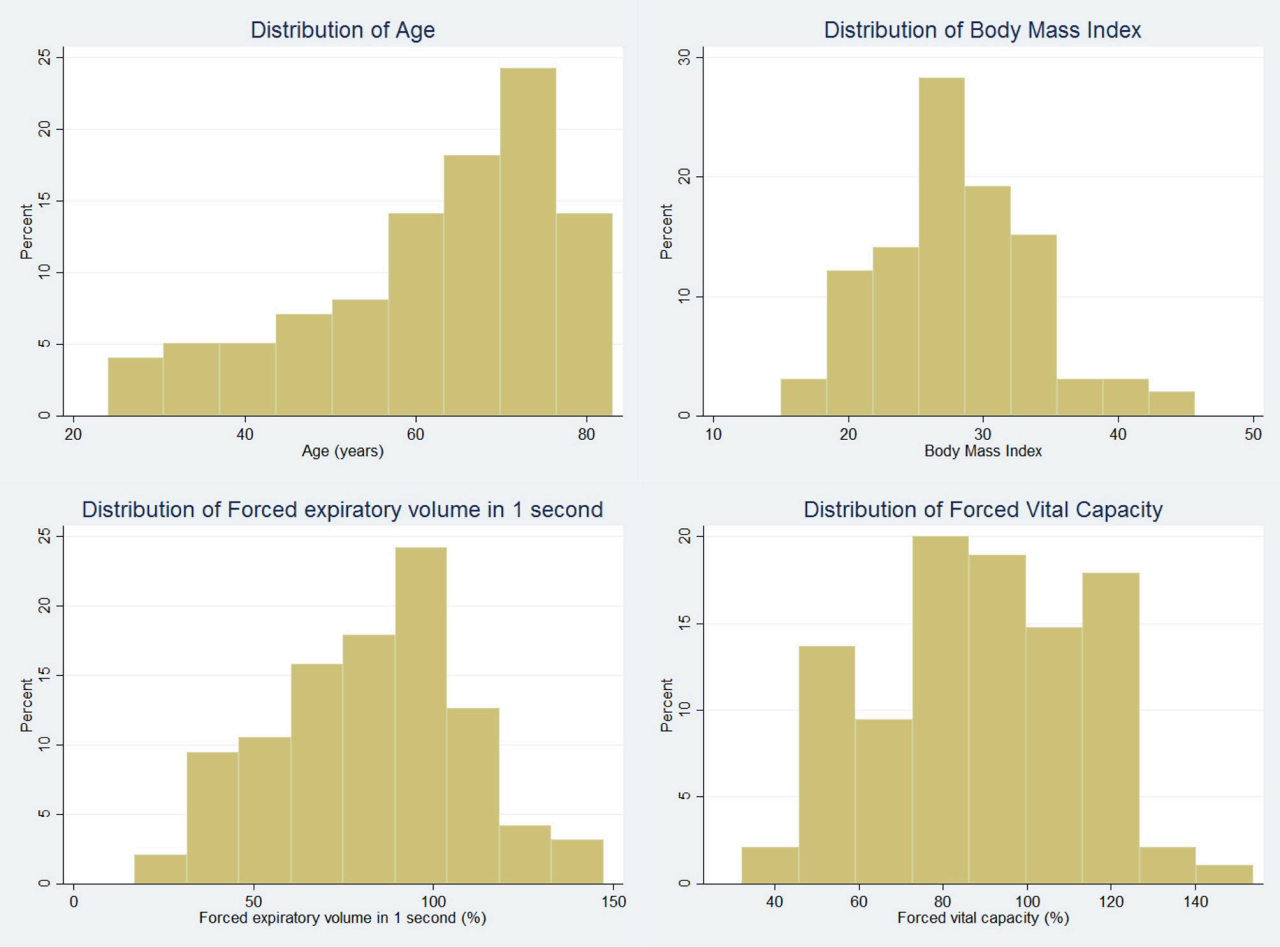
**Histogram demonstrating distribution of patient variables**.

One hundred consecutive patients (47 male) were recruited with a mean age of 62.1 years. Thirty-seven patients had obstructive lung diseases including asthma and Chronic Obstructive Pulmonary Disease (COPD), 42 patients had interstitial lung diseases. The remaining 21 patients had not been diagnosed with respiratory disease. Mean FVC was 89.2% predicted (range 30.1–156.4%), mean FEV1 was 81.9% predicted (range 23.8–155.5%), with a mean FEV1/FVC ratio of 0.74 (range 0.36–0.99). Demographic details are shown in Table 1 and distribution of age, body mass index (BMI), FEV1, and FVC in Figure 4.

**Table 1:** **Subject characteristics**.

	**Mean/Number**	**Standard deviation/%**
Age (Years)	62.1	14.7
Male (n)	47	47.5
Body mass index (kg/m^2^)	28.2	5.7
**DIAGNOSIS**		
Obstructive lung disease	37	37.4
Fibrotic lung disease	42	42.4
Other	20	20.2
FVC, L (% predicted)	2.99 (89.2)	1.20 (25.3)
FEV1, L (% predicted)	2.17 (81.9)	0.99 (27.2)
FEV1/FVC ratio	0.737	0.131

*FVC, forced vital capacity; FEV1, forced expiratory volume in 1 s*.

Eight of the first 11 subjects were removed due to technical issues with the camera preventing data from being usable. At this point a pressure-sensing buzzer was introduced to assist subjects in maintaining their posture according to guidelines. After this modification, data were unusable for only 6 of the subsequent 89 (6.7%). Data were discarded if patients were unable to perform spirometry (*n* = 3), movement artifact prevented accurate model estimate generation (*n* = 1) or technical issues prevented data from being recorded (*n* = 2). Two hundred and fifty one efforts were recorded for forced spirometry maneuvres, 280 for relaxed maneuvres including vital capacity and inspiratory capacity.

### Performance of model

**Figure 5:**
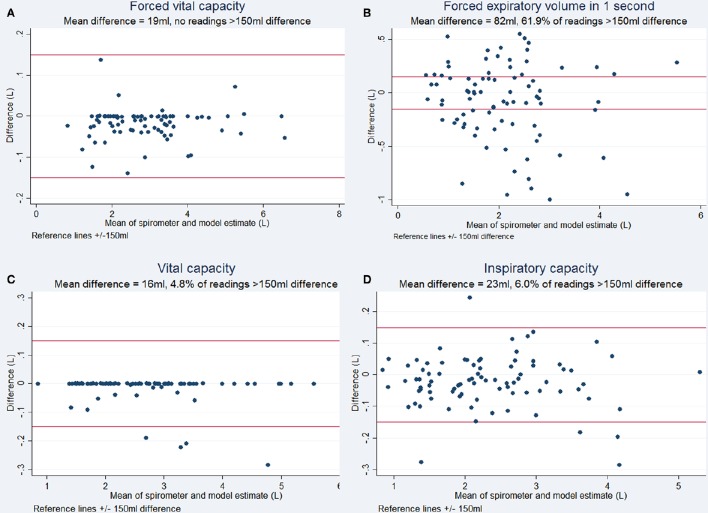
**Bland Altman plots for respiratory maneuvres demonstrating the degree of agreement between the volume estimated by the Kinect to the volume measured by spirometry (Bland and Altman, 1999)**. **(A)** Forced Vital Capacity, **(B)** Forced Expiratory Volume in 1 second, **(C)** Vital Capacity, and **(D)** Inspiratory Capacity. ±150 ml has been used as limits of acceptable difference, according to accepted criteria (Miller et al., 2005).

Respiratory rate tracking with normal tidal breathing using the Kinect agreed with the respiratory rate calculated from spirometry traces (Figure 3). The estimates generated by the 3D model correlated highly with spirometric values for FVC (*r* = 0.999, *p* < 0.001), FEV1 (*r* = 0.937, *p* < 0.001), VC (*r* = 0.998, *p* < 0.001), and IC (*r* = 0.995, *p* < 0.001), demonstrating their linear relationship. The chest volume change estimated by the Kinect camera during spirometry tracked respiratory rate accurately and estimated FVC and vital capacity to within ± <1%. Forced expiratory volume estimation did not demonstrate acceptable limits of agreement, with 61.9% of readings showing >150 ml difference. Bias between techniques for FVC was 19 ml (SD 37 ml), for VC 16 ml (SD 51 ml), for IC 23 ml (SD 81 ml). Variability and bias were greater for FEV1 (82 ml, SD 363 ml). Bland-Altman plots are shown in Figure 5.

Within subject variability in measurement was assessed for both spirometry recordings and 3D model for simultaneous measurements. The 3D model showed acceptable within subject variability, with high correlation to spirometry variability for FVC (2.9%, *r* = 0.862), VC (4.5%, *r* = 0.869), and IC (5.7%, *r* = 0.825). Within subject variability was higher, with less robust correlation for FEV1 (10.7%, *r* = 0.177).

### Regression analysis

Linear regression analysis was performed to identify variables predictive of magnitude of error in estimation of FEV1, FVC, VC, and IC. Variables included in the model were age, gender, BMI, pack years smoking, diagnosis, and testing date, in addition to the appropriate lung volume measurement. No variables were predictive of error for all measurements. Higher discrepancy between spirometry and camera model estimates were seen at greater values of FEV1 (FVC discrepancy *r* = 0.418, *p* < 0.01, FEV1 discrepancy *r* = 0.370, *p* < 0.01).

When spirometry results were classified into abnormal and normal categories by the criterion of FEV1/FVC ratio < 0.7, 30 of the 84 patients had obstructive spirometry. The camera model estimate correctly classified 25 readings as abnormal and correctly classified 37 readings as normal. This gives a positive predictive value of 0.833 and a negative predictive value of 0.685.

### Scaling factor analysis

Scaling factors were calculated for each spirometry effort to relate this to its associated camera model estimation. Initial regression analyses demonstrated heteroscedascity with untransformed scaling factors. This was reduced by log-transforming the scaling factors. The results from multivariable linear regression analysis of these log-transformed factors separately for relaxed and forced spirometry maneuvres are shown in Tables 2, 3. The multivariable model for forced maneuvres contained age, gender, height, weight and pack years of smoking. This model explained 37.0% of the variance in the scaling factor [*F*_(5, 245)_ = 28.77, *p* < 0.0001]. A similar model for relaxed maneuvres explained 50.5% of the variance in the scaling factor [*F*_(5, 274)_ = 55.92, *p* < 0.0001].

**Table 2:** **Multivariable linear regression of scaling factors for forced spirometry maneuvres**.

	**Coefficient**	**Significance**
Age	−0.002	0.184
Gender	−0.285	<0.001
Height	0.013	0.001
Weight	0.002	0.147
Pack years	0.005	<0.001
Constant	−13.981	<0.001

**Table 3:** **Multivariable linear regression of scaling factors for relaxed spirometry maneuvres**.

	**Coefficient**	**Significance**
Age	0.005	<0.001
Gender	−0.479	<0.001
Height	0.003	0.344
Weight	0.004	<0.001
Pack years	0.002	0.042
Constant	−12.374	<0.001

## Discussion

This preliminary assessment of a low cost, “off-the-shelf” game console camera to estimate chest volume changes during spirometry suggests it can track respiratory rate and motion accurately. Currently this technology is limited by the need to generate a scaling factor for each subject in order to convert the volume change estimated in the model to accurate airflow. Positive predictive value for obstructive spirometry was 0.833, however the negative predictive value of 0.685. This technique may still have potential, if further refined, as a method of screening for respiratory disease.

This is the largest study of its kind, conducted in a “real world” clinical setting. The study population includes a wide variety of commonly encountered lung pathologies and a broad spectrum of spirometric values, age and body habitus, making our findings relevant, and generalizable.

Using this modeling approach, FVC and VC estimates can be highly accurate, however there were significant errors in estimates of FEV1 according to Bland-Altman analysis, despite very strong correlation. One explanation for this discrepancy is the reliance of the computation on the “key points” directly related to VC and FVC from the spirometric volume-time curves. In contrast the FEV1 is not directly related in the same way to the algorithm from which volume estimates are generated. The inaccuracy of the FEV1 preclude its use in quantitative spirometry currently.

Other approaches to remote chest volume change estimation have included optoelectronic plethysmography, using light-based tracking of multiple markers placed on the body. This approach has demonstrated similarly close correlation with spirometry in 10 healthy subjects with normal lung function (Aliverti et al., 2001), however this was limited by the requirement for multiple chest wall markers. Our technology does not require any additional components sited on the subject.

Structured light plethysmography is the most progressed of remote lung function assessment techniques. Initially developed in the 1980s, commercially available devices are now available such as the Thora3Di (Pneumacare, Cambridge, UK). These rely on the projection of a grid pattern of light recorded by two cameras from which volume changes are estimated, following a scaling process analogous to that described here. Studies published in abstract form have been from healthy subjects with normal lung function, showing good correlation with spirometry and acceptable variability by Bland-Altman analysis (Lau et al., 2010). While similar to our technology, the recording device for the Thora3Di is significantly larger and more expensive. The Microsoft Kinect is an “off the shelf” readily available product on the high street, with no hardware development required. We anticipate a wide range of uses for the Kinect-based approach, in particular the potential to support patients and healthcare professionals in the home environment. Benefits of the technique reported in our study include simplicity of set-up with only a single camera required, no requirement for specialist training and low cost. This needs to be assessed in appropriate settings as the technology is developed. The experimental set-up used here must be adapted to use in, for example, home monitoring.

LIDAR technology, in the form of time-of-flight cameras, has been examined in healthy subjects using the Kinect V2 depth sensor by other groups. This technology has been used to assess the severity of airflow obstruction, using 14 healthy subjects blowing down a series of straws of diminishing diameter while lying supine (Ostadabbas et al., 2015). The authors report 76.2% accuracy in predicting three levels of severity of airway obstruction. A further study investigated the correlation of time-of-flight estimated breathing signals with those obtained by inductance plethysmography, observing *R*^2^-values of 0.85 for chest movement and 0.91 for abdominal movement. These approaches are similar to our own, with differences in the algorithms underlying volume estimation. They support the potential of Kinect-based technology in the accurate estimation of lung volumes and airflow, including obstruction.

## Next steps in development

This technology is currently reliant on individual scaling factors to estimate lung volumes. Work is currently ongoing to calibrate the camera model using one spirometry effort and incorporate demographic details (which appear to predict around 50% of the variance factor) to compute a scaling factor to be applied to future efforts.

It is also possible that with the addition of second Kinect camera behind the subject the accuracy of the model could be improved by compensating for movement artifact and improved detection of posterior chest wall movement. This may also enable estimation of lung volumes currently reliant on plethysmography.

We have shown that this technology can accurately detect respiratory rate and changes in lung volumes, it can also be used to collect data in real-time. This makes it particularly appealing for use in home monitoring of respiratory disease. The technology must, however be adapted and refined for use in a home setting from its current experimental set-up. This is an area for development and assessment. Future research questions include; which groups of patients would benefit most from this technology? It could be anticipated that patients with frequent exacerbations of respiratory disease, those with high healthcare usage or patients at high risk of deterioration, such as those awaiting lung transplantation could benefit from closer monitoring to enable early intervention in the event of decline. This will require comprehensive study once the technology has been refined further.

## Future applications

This study highlights the potential of 3D chest wall motion tracking in respiratory medicine. In the short to medium term, this technique could be used to remotely monitor respiratory rate, screen for abnormal spirometry or assist patient/ventilator synchronization. With modest further refinement it could also be used to examine regional chest wall movement in more detail (e.g., for the detection of pleural pathology such as effusion and pneumothoraces). This technology has the potential to be implemented in both inpatient, clinic and home settings.

Looking more broadly, it is clear that healthcare systems require significant redesign to meet escalating demand and focus away from a reactive, unplanned (and expensive) hospital based system toward community based chronic disease management systems. These changes will be reliant to a large extent on the adoption of novel and clinically relevant sensors of respiratory function, as presented here, in addition to other ways of monitoring physical activity and air pollution for example. The “Internet of Things” (IoT), where every-day objects are connected to the internet and communicate with each other, and “big data” utilizing large amount of information will enable these sensors to all be connected and generate continuous data to build a highly detailed picture of health status at home.

As clinicians we understand that each individual patient's reasons for admission to hospital and deterioration in health are complex—projects such as SPHERE are working to capture and understand this complexity and use it to support the patient and healthcare workers to improve disease outcomes. However, it is vital that clinicians engage with engineers, healthcare administrators, computer scientists and others to ensure that we focus on important clinical problems.

## Conclusions

A 3D “time of flight” camera is a novel and promising approach to accurately track chest volume changes which has the advantages of cost and ease of setup. While it has limitations in quantifying lung volume changes, it has a wide range of other potential applications which merit exploration. This is an example of productive, collaborative efforts between clinicians and other disciplines to develop new technologies to advance the field of respiratory physiology assessment.

## Author contributions

JD and MM conceived the study. CS, VS, and JV collected the data. Model construction was performed by VS, SH, MM, MC, and DD. Data analysis was performed by CS, VS, and SH. The manuscript was written by CS and JD. All authors reviewed and approved the manuscript.

## Funding

VS has been supported by University of Bristol Alumni Foundation. This work was made possible by the EPSRC award to the SPHERE project.

### Conflict of interest statement

The authors declare that the research was conducted in the absence of any commercial or financial relationships that could be construed as a potential conflict of interest.
